# The leaky pipeline in research grant peer review and funding decisions: challenges and future directions

**DOI:** 10.1007/s10734-020-00626-y

**Published:** 2020-10-03

**Authors:** Sayaka Sato, Pascal Mark Gygax, Julian Randall, Marianne Schmid Mast

**Affiliations:** 1 Department of Psychology, University of Fribourg, Rue P. -A.-de-Faucigny 2, CH-1700 Fribourg, Switzerland; 2 Research Promotion Service, University of Fribourg, Fribourg, Switzerland; 3 Faculty of Business and Economics, University of Lausanne, Lausanne, Switzerland

**Keywords:** Gender inequality, Research grant funding decisions, Equal opportunities, Female researchers, Grant peer review

## Abstract

The growing literature on gender inequality in academia attests to the challenge that awaits female researchers during their academic careers. However, research has not yet conclusively resolved whether these biases persist during the peer review process of research grant funding and whether they impact respective funding decisions. Whereas many have argued for the existence of gender inequality in grant peer reviews and outcomes, others have demonstrated that gender equality is upheld during these processes. In the present paper, we illustrate how these opinions have come to such opposing conclusions and consider methodological and contextual factors that render these findings inconclusive. More specifically, we argue that a more comprehensive approach is needed to further the debate, encompassing individual and systemic biases as well as more global social barriers. We also argue that examining gender biases during the peer review process of research grant funding poses critical methodological challenges that deserve special attention. We conclude by providing directions for possible future research and more general considerations that may improve grant funding opportunities and career paths for female researchers.

## The leaky pipeline in research grant peer review and funding decisions: challenges and future directions

Academia is by no means an exception when considering the matter of gender inequality extant in the current labour market. Despite years of continuous effort, the European average in 2016 indicates that female academics continue to be underrepresented in academia. Specifically, among all research positions held by women in Europe, only 7.4% hold the highest research positions^1^ as opposed to 16.7% for men (She Figures; European Commission, 2018). Nonetheless, the gender issue is not limited to a smaller number of female professors. A series of aspects differ among female and male researchers that may be responsible for women not wanting to pursue an academic career or for women not advancing at the same pace and with the same success in their academic careers. The present paper highlights these aspects, focusing particularly on issues relating to research grant review and funding decisions that may disadvantage women in their academic careers.

Recent studies examining women’s careers in academia report that the indices of academic excellence such as employment (Clauset et al., 2015; Moss-Racusin et al., 2012), pay grade (e.g. Barbezat & Hughes, 2005; C. B. Travis et al., 2009), performance evaluation (e.g. Madera et al., 2009; Rubini & Menegatti, 2014; Storage et al., 2016), authorship (e.g. Macaluso et al., 2016; West et al., 2013), peer-review opportunities (e.g. Lerback & Hanson, 2017), attainment of research grants (e.g. Bedi et al., 2012; van der Lee & Ellemers, 2015), and tenure (e.g. Perna, 2001; Weisshaar, 2017), all testify to a more challenging reality for female researchers and their careers than for male researchers. Importantly, these indices of achievement are not independent of each other in an academic career. Instead, they work cumulatively, gradually constructing a researcher’s profile as the individual advances through their career (e.g. Bol et al., 2018). For example, lacking sufficient first-authored papers can lead to low perceived scientific contributions (Wren et al., 2007), which, in turn, may imply obtaining less research funding and subsequently leading to fewer research activities overall (Larivière et al., 2011). The vicious circle often continues, with fewer research activities resulting in fewer publications (Gillett, 1991), and further diminishing the chances of developing an excellent professional profile (Liner & Sewell, 2009). Success in each of these milestones is imperative for progress in an academic career, where weaknesses in one or more may have a detrimental impact on the career as a whole. In this respect, the mentioned studies showcase the systematic disadvantage female researchers are confronted with at every stage of their career development. As the oft-cited metaphor of the ‘leaky pipeline’ suggests, these potential difficulties lead to a large number of female researchers opting out of science at early stages of their careers (Martinez et al., 2007). A better understanding of how women are disadvantaged and confronted with greater career challenges in academia is, thus, key in providing insight into how academia can better retain female researchers and assure that their careers advance comparably to those of their male counterparts.

In the present paper, we explore the particular challenges that women face in obtaining extramural research funding, a fundamental index of a successful research career (e.g. Archer, 2008; Hornbostel et al., 2009; Sutherland, 2017 ; van den Besselaar & Sandström, 2015). Obtaining grant funding not only supports one’s research activities, but it is often taken as an indicator of a researcher’s productivity as well as their competence (e.g. Archer, 2008; Hornbostel et al., 2009; Sutherland, 2017; van den Besselaar & Sandström, 2015), and is directly related to securing subsequent promotions and tenure opportunities (e.g. Gerritsen et al., 2013; Liner & Sewell, 2009; Schimanski & Alperin, 2018; van den Besselaar & Sandström, 2015). However, unlike other academic assessments such as article peer reviews, grant funding reviews are rarely blind (Sigelman & Scioli, 1987), paving the way for reviewer biases to take effect. Reviewers might favour funding applications by male researchers over those of female researchers, even implicitly so. Considering such potential disparities in the treatment of women and men in grant funding review is central for understanding differences in female and male career trajectories. To this end, we provide a general synthesis of the representative literature that indicate the existing issues linked to this potential gender bias. Rather than presenting an exhaustive review of the existing literature, we aim to provide critical considerations on how contradictory research findings can be approached to better account for the intricate impact of gender in academia.

In the following, we first present the mixed findings in the research that assesses whether applicants’ gender can be identified as a risk factor in the evaluation process in research grant funding^2^. We particularly cover a wide range of funding schemes in countries of North America, Western Europe, and Australia where high impact research output is considered to be central (Compendium of bibliometric scientific indicators 2014). Second, we examine the potential reasons for the divergent findings by identifying the sources of gender discrimination in academia and the variation in methodology observed across studies. In doing so, we aim to highlight the bigger social context from which gender inequalities emerge and directly impact women’s research careers.

## Is gender a risk factor for the funding peer review process and grant funding decisions?

The process of evaluating a research grant application is largely based on peer review, in which several expert reviewers assess a given proposal on its scientific quality (e.g. in terms of novelty and pertinence) and the applicant’s academic profile (Marsh et al., 2008). The process is targeted at gaining specialised evaluations of potentially high-quality research and making informed decisions on how research funding should be allocated (Sandström & Hällsten, 2008). Although peer review is largely considered indispensable for academic gatekeeping, this evaluation procedure has nonetheless been criticised for its lack of reliability (Cicchetti, 1991; Marsh et al., 2008) and reviewer agreement (Pier et al., 2018). In other words, funding decisions may not necessarily be explained by the quality of the applicant profile and their scientific proposals alone.

This potential problem has led to a growing concern that the grant peer review process may have a subjective component (Pier et al., 2018), wherein funding decisions can be impacted by reviewers’ individual biases, along with the systemic biases extant in academia (Witteman et al., 2019). Because the reviewers are not blind to the identity of the applicants (Sigelman & Scioli, 1987), their decisions can be largely influenced by the various information available to the reviewer, including the applicant’s race (Ginther et al., 2012), their affiliated institutional size (Murray et al., 2016), institutional prominence (Ceci & Peters, 1982), and school of thought (G. D. L. Travis & Collins, 1991). As the well-documented tendency to undervalue women’s scientific achievements known as the Matilda effect suggests (Rossiter, 1993), it seems reasonable to assume that gender stereotypes may also affect peer evaluations for fellowships and grants.

One of the first papers to portray potential gender discrimination in the process of grant peer review was the seminal study published by Wennerås and Wold (1997) in *Nature*. Based on an investigation of postdoctoral fellowships at the Swedish Medical Research Council, the study provided evidence to suggest that applicants’ gender reliably predicted the reviewers’ competence scores that were allotted to them. This was true even when taking into account applicants’ productivity scores consisting of multiple bibliometric measures, such as their first and last authored publications or the impact factor of the journal in which they published. Female applicants were found to receive lower evaluations than male applicants and were required to be 2.5 times more productive than the average male applicant to receive the same scores in their review evaluations. Although this exposure of gender inequality in grant peer review initially shook academia, over the years, their empirical claim has been met with a significant amount of disagreement, namely, being criticised for their statistical analyses. For instance, it has been pointed out that multiple variables reflecting applicants’ productivity (e.g. number of publications, impact factor) were entered individually into separate regression models to predict reviewers’ scores. However, fitting these variables into a single rather than separate models would have been more relevant to reflect the real world more accurately (see Ceci & Williams, 2011), as in recent studies on grant funding (e.g. Tamblyn et al., 2018).

Replication attempts to reproduce Wennerås and Wold’s (1997) effects quickly followed, with the study becoming a major catalyst for igniting the discussion on male favouritism in the decisions linked to research grant funding. These replication attempts, however, have since generated mixed results as to the direct impact of applicants’ gender on grant peer review and funding decisions, with studies on one side of the argument claiming that the process of grant peer review and decisions are dependent on applicant gender, whereas the opposing side have argued for the absence of any gender discrimination.

## The current state of research

Studies that have found gender inequalities show that applicant gender plays a focal role in reviewer decisions, a finding that has been established across numerous disciplines (e.g. dermatology research: Cheng et al., 2016; global infectious disease research: Head et al., 2013; cognitive science: Titone et al., 2018; cancer research: Zhou et al., 2018) and within various national contexts (e.g. UK: Blake & La Valle, 2000; Netherlands: Brouns, 2000; van der Lee & Ellemers, 2015; USA: Eloy et al., 2013; Jagsi et al., 2009; Australia: Over, 1996; Sigelman & Scioli, 1987; Canada: Burns et al., 2019; Tamblyn, et al., 2018; Switzerland: Severin et al., 2019). Female applicants have been shown to be disfavoured compared to their male counterparts, resulting in, proportionally, fewer of their studies being funded (Gannon et al., 2001; Head et al., 2013; Jagsi et al., 2009; Steinþórsdóttir et al., 2019; Zhou et al., 2018), fewer requests being awarded (Waisbren et al., 2008), and lower funding amounts being allotted (Bedi et al., 2012; Eloy et al., 2013; Steinþórsdóttir et al., 2019; Zhou et al., 2018). Many of these studies have looked at individual grant schemes in specific disciplines and/or under particular evaluation criteria, therefore characterising precise conditions in which gender inequalities may take form.

A crucial point to note is that these studies show that the effects of gender can persist even when relevant causal variables relating to the evaluation are taken into consideration. These include, among others, factors such as applicant productivity, discipline, and academic seniority. In other words, even when women and men’s productivity indices such as bibliometric measures or their discipline were kept constant, these gender disparities persisted. A recent study by Tamblyn et al. (2018), for instance, showed that female applicants were found to receive consistently lower reviewer scores than male applicants for grant schemes submitted to the Canadian Institutes of Health Research even after the authors controlled for the applicant’s scientific productivity along with other potentially influencing variables (e.g. applicant age, reviewer characteristics). This female disadvantage was further supported by a meta-analysis conducted by Bornmann et al. (2007) on 21 studies published between 1987 and 2005, indicating that men had 7% greater odds of obtaining funding than women.

Despite the accruing evidence in favour of this gender divide in the grant peer review process and funding decisions, the effect is yet to be systematically demonstrated. In opposition to the studies indicating that women are consistently undervalued during their application evaluations, a growing literature has in fact argued for the absence of gender differences, asserting that women and men have relatively equal chances to obtain grant funding in different academic disciplines and countries (e.g. Belgian Fonds de la Recherche Scientifique: Beck & Halloin, 2017; NIH: Forscher et al., 2019; Kalyani et al., 2015; Pohlhaus et al., 2011; Warner et al., 2017; Medical Research Council of Canada: Friesen, 1998; Wellcome Trus: Grant et al., 1997; Swiss National Science Foundation: Reinhart, 2009; National Health and Medical Research Council of Australia: Ward & Donnelly, 1998; RAND Corporation, 2005). Contrary to many of the aforementioned studies substantiating gender effects in specific grant schemes and contexts, these findings have been obtained from studies examining large-scale data ensuring sufficient effect sizes and generalisability (Jayasinghe et al., 2003; Marsh et al., 2008, 2011). In fact, by revisiting Bornmann et al. (2007) aforementioned highly recognised meta-analysis, Marsh et al. (2009) suggested that the reported gender bias was modest and statistically negligible, demonstrating that their results were not tenable. Moreover, some studies have even reported an opposite bias, indicating that women may be favoured over men (Marsh et al., 2009; Sandström & Hällsten, 2008).

Studies attesting equal opportunities have shown that female and male applicants show comparable proportions in approval rates (Beck & Halloin, 2017; Boyle et al., 2015; Friesen, 1998; Kalyani et al., 2015; Ward & Donnelly, 1998), funding amounts (Beck & Halloin, 2017; Boyle et al., 2015), and attributed reviewer scores (Forscher et al., 2019). For instance, Grant et al. (1997) employed a similar approach to the Wennerås and Wold (1997) study to investigate applications submitted to the Wellcome Trust and the UK Medical Research Council. The study showed that funded rates between women and men, as well as their publication counts, were equal, further justifying that funding success was in line with the applicants’ productivity. More convincing evidence has been reported from controlled experiments where the effect of applicant gender was investigated by simulating an actual grant review process. Forscher et al. (2019), for example, manipulated the gender of the applicants’ names on previously submitted NIH proposals to examine whether the initial round of reviewer evaluations was dependent on applicant gender or race. Researchers participating as reviewers submitted their reviews. The study revealed no impact of gender on the first round of NIH reviews, concluding that applicants were equally treated in their funding decisions.

## Potential sources and reasons for the mixed findings: identifying the source of gender discrimination

In light of the developing research, how, then, can we reconcile the emergence of such opposing views? We argue that a point of departure to such a question is to first identify the potential sources and causal relations that may lead to potential gender inequalities.

### Individual and systemic biases

Witteman et al. (2019) point to individual bias and systemic bias as key aspects that can account for why women may be disfavoured in grant peer review processes and funding decisions. The first explanatory aspect, *individual bias* refers to reviewers’ conscious and unconscious stereotypical biases emerging during the actual grant peer review process. These biases typically assume women to be less competent than men and can lead to less favourable evaluations for women. Van der Lee and Ellemers’ (2015) study is a case in point. In their study, female applicants submitting to the Netherlands Organisation for Scientific Research were less likely to be prioritised than male applicants on the evaluation of ‘quality of researcher’, although evaluations for their ‘quality of proposal’ and ‘knowledge utilisation’ were found to be as equally strong as those of their male counterparts. Similar results were also reported by Witteman et al. (2019) in their investigation of funding programmes and their comparison of different evaluation criteria at the Canadian Institutes of Health Research. As these studies examined specific assessment criteria of the peer review process rather than simple outcome data (e.g. funding totals, funding decisions), they speak to the fact that a grant application may be disregarded by reviewers simply because the applicant is a woman, even if her track record and skill sets are considered to be of equal or even better quality than that of male applicants.

These individual biases, emerging during the peer review process, are considered to be shaped by stereotypical beliefs in academia about the traits that constitute a skilled researcher. From a social psychological perspective, people possess both descriptive and prescriptive stereotypes, where the former refers to the belief about how women and men commonly act, and the latter to the belief about how women and men *should* act (Cialdini & Trost, 1998; Fiske, 1998). According to the Social Role Theory (Eagly et al., 2000; Eagly & Steffen, 1984), many of these gender stereotypes develop as a result of socialisation, in which women and men learn and develop their roles based on the division of labour in society and social expectations. As a result, we commonly associate women with communal traits (e.g. *friendly*, *unselfish*, *emotionally expressive*) and men with more agentic traits (e.g. *competent*, *assertive*, *independent*) (Eagly & Karau, 2002). Within the context of academia, such agentic traits are considered to be assets to pursue scientific competition and rigour. As a corollary, if reviewers unconsciously or even consciously apply prescriptive stereotypes during the review process, expecting applicants to possess agentic traits while also holding general descriptive stereotypes that assume gender-specific characteristics, these preconceived beliefs or preferences can potentially provide a source for more critical or biased evaluations for female researchers (e.g. Carli et al., 2016; Madera et al., 2009). Indeed, research has shown that reviewers prefer using wording associated with male-stereotyped traits (e.g. *challenging*, *independent*) rather than female-stereotyped traits (e.g. *responsible*, *thorough*) in their written communications during the application review process, a finding which has been taken to reflect the endorsement of masculine traits that superior applicants are expected to possess (van der Lee & Ellemers, 2015). The concern, however, is that reviewers are more likely to allocate these agentic traits (e.g. *daring*) as well as standout words (e.g. *amazing*) to male than female applicants in their review, as they often assume that men possess traits better suited to pursuing quality research (Kaatz et al., 2015, 2016; Magua et al., 2017). Consequently, women who are considered to lack the ‘necessary’ agentic traits to succeed in academia may be perceived as being less fit to be the ideal candidate in spite of their proven academic capabilities (Heilman, 1983), further contributing to their harsher treatment overall.

An issue worth noting is that the emergence of these individual biases is independent of any specific reviewer characteristics. For instance, despite there being fewer female reviewers involved in the peer review panel given that its composition generally reflects the academic demographic, reviewer gender has not been shown to contribute to any gender discrimination against applicants (Bornmann & Daniel, 2007; Jayasinghe et al., 2001, 2003). Some studies have also shown that male reviewers were prone to give, overall, higher evaluations than female reviewers (e.g. Broder, 1993; Jayasinghe et al., 2003; Severin et al., 2019), although this effect was found to be rather modest (Severin et al., 2019). Additionally, the common assumption that external reviewers may hold preferences for applicants with the same or a specific gender (Jayasinghe et al., 2003) has been examined in grant schemes for the Australian Research Council (Jayasinghe et al., 2001, 2003; Marsh et al., 2008, 2011), the Austrian Science Fund (Mutz et al., 2012), the German Boehringer Ingelheim Fonds (Bornmann & Daniel, 2007), and the Swiss National Science Foundation (Severin et al., 2019), and none have substantiated the effect. Thus, no interaction effects between applicant gender and reviewer gender have been found thus far (Bornmann & Daniel, 2007; Jayasinghe et al., 2001; Marsh et al., 2011; Mutz et al., 2012), with these results being considered as generalisable across disciplines (Marsh et al., 2008, 2011).

*Systemic bias*, the second explanatory aspect suggested by Witteman et al. (2019), refers to grant scheme design and grant evaluation criteria that unequivocally place men in favourable positions. In other words, whereas gender in itself may not lead to disadvantages that can be detrimental to men’s careers, women are more directly at risk of being discriminated against based on their gender. For example, compared to men, women are less likely to have their work cited (Bendels et al., 2018; Caplar et al., 2017; Maliniak et al., 2013), to author publications (Jagsi et al., 2006; Larivière et al., 2011), to be accepted to conferences (Roberts & Verhoef, 2016), and to take the more prestigious first and last author positions of a publication (West et al., 2013). These challenges also become evident in the workplace, where women are employed in unstable, intermediary positions, such as assistant professor positions, more often than men. In these positions, women are required to teach more (e.g. Gibney, 2017; Link et al., 2008; Winslow, 2010) and work longer office hours (e.g. El-Alayli et al., 2018), making it difficult to carry out their research activities. Additionally, women also face unfair limitations with respect to institutional resources, receiving less lab space, fewer assistants, and lower travel budgets than those of men (Johns Hopkins University Committee on the Status of Women, 2006; Massachusetts Institute of Technology, 1999). These disadvantages inevitably make it difficult for female researchers to thrive in their careers and facilitate men to purse their career. In the context of research grant peer review and funding decisions, these systemic disadvantages that women face accumulate, resulting in lower productivity, and hence, a weaker profile that may not be sufficient for the grant scheme criteria. In contrast, male researchers are not faced with comparable challenges and are therefore able to maintain a stronger bibliometric impact that is stable across age and discipline (Larivière et al., 2011).

Despite these noticeable challenges, there are, however, certain disciplines—such as the social sciences—where equality is better upheld given that women have a more equal role to men (Boyle et al., 2015). Other disciplines, such as STEM fields (i.e. science, technology, engineering, and mathematics), remain generally male-dominated, where women are less likely than men to opt for an academic degree, as well as to choose and remain in a related occupation (Cheryan et al., 2017). The relative absence of women in the STEM fields thus produces a shortage of female role-models and networks, where women are required to comply with the discipline’s masculine culture to advance into their careers (Hart, 2016). Grant funding differences between women and men across distinct disciplines prominently reflect these characteristics. This is exemplified in countries such as Iceland (Steinþórsdóttir et al., 2019), where gender distribution is generally equal: women and men globally show comparable success in their granted amounts and numbers, particularly in the social sciences, whereas women are more likely to receive higher grants than men in female-dominated fields like education, and men are substantially more likely to be awarded grants and receive higher amounts of funding in male-dominated fields such as engineering, natural sciences, health sciences, and humanities.

These key aspects highlight that gender inequalities cannot be attributed solely to the gender bias emerging *during* the grant funding peer review process. Rather, they may also result from the cumulative discrimination and challenges that women endure given the academic culture. Put differently, women bear the burden of discriminatory reviewer biases that identify them as being less competent than their male counterparts even if all other indices, such as their skill sets, are considered equal. At the same time, women may face other challenges over the course of their career that derive from the male-dominated culture of academia which results in women’s applications being less effective than those of male applicants.

### Social barriers

In line with the aforementioned biases, *social barriers* may also lead to disadvantages for women’s careers. For example, family obligations, which in androcentric cultures place women in charge of the home and family (e.g. Gutek et al., 1981), assign women additional burden outside of the workplace. In fact, there is preliminary evidence that the COVID-19 pandemic also disproportionally puts female scientists at a disadvantage with comparatively fewer publications submitted during lockdown by female as compared to male researchers (Frederickson, 2020). Essentially, bearing and caring for children has been shown to have a greater impact on women’s academic careers than that of men (Hunter & Leahey, 2010), restricting women’s working conditions and time (Ledin et al., 2007). These circumstances inevitably cause women a consistent lack of productivity throughout their careers (Cole & Zuckerman, 1984), reducing their academic impact.

This lack of women’s productivity subsequently promotes the assumption that female researchers are less competent, affecting their teaching assessments (Boring et al., 2016; MacNell et al., 2015; Mengel et al., 2019) and competence evaluations (Moss-Racusin et al., 2012) from both students and colleagues. Consequently, these constraints hinder women’s representation in academia and facilitate the preservation of a general male-dominant climate. For these reasons, women generally do not choose to remain in academia, with many opting out during the transition period from postdoctoral to secure faculty positions (Ley & Hamilton, 2008). However, in situations where women do decide to remain, they are nonetheless shown to be less inclined to apply for promotions (Pyke, 2013), leaving senior researcher positions underrepresented by women (Ley & Hamilton, 2008; Pohlhaus et al., 2011).

In the context of grant funding, this situation creates a general shortage of women in the academic workforce and, importantly, female senior faculty who make up the cohort of eligible applicants (Holliday et al., 2014). This insufficient number of eligible women inevitably reduces the number of applications from female candidates, which may directly explain why women are found to be less successful in obtaining research grant funding. In fact, it has been shown that when women attain senior positions, they are as successful as men in obtaining funding (Holliday et al., 2014). To illustrate this, Waisbren et al. (2008) demonstrated significant gender differences in measures such as application success rate, submission rate, and the number of funding years requested for grant applications submitted by faculty at several Harvard Medical School-affiliated institutions. However, when the authors controlled the academic rank of the applicants, success rates, but not submission rates no longer showed significant differences, indicating a strong explanatory content of academic seniority on grant funding success.

In addition to the insufficient number of eligible senior researchers, women, compared to men, are generally less inclined to apply for grant funding or renewal (Beck & Halloin, 2017; Blake & La Valle, 2000; Boyle et al., 2015; Friesen, 1998; Grant et al., 1997; Jayasinghe et al., 2003; Marsh et al., 2008; Pohlhaus et al., 2011; Severin et al., 2019; Ward & Donnelly, 1998). On a practical level, institutional support may not be available for eligible women to relieve them of administrative or teaching duties to prepare for the grant writing process or to support them in receiving the necessary training needed for preparing applications (Easterly & Pemberton, 2008). On a more psychological level, female researchers are also more likely to downgrade their own competence than male researchers (Easterly & Pemberton, 2008; Gordon et al., 2009), which can discourage them from pursuing proactive grant submission behaviour.

These issues shed light on the fact that beyond applicant gender, there are other contextual variables, such as family and academic challenges, that play an influencing role on women’s careers and that it is imperative to address these extraneous variables when considering research grant funding. Discounting such variables can potentially cause statistical complications known as Simpson’s Paradox where an assumed trend between two variables is equally dependent on a third variable and individually examining them can result in revealing an opposite trend from what was initially shown (Simpson, 1951). This was precisely highlighted by Volker and Steenbeek (2015) and Albers (2015), in response to the aforementioned van der Lee and Ellemers (2015) study demonstrating gender differentials in funding success. Specifically, their study was criticised for not taking into account other variables, such as the acceptance rate within each sub-field given that the female applicants in their study were more likely to apply for competitive fields (e.g. the medical and social sciences) and male applicants for less competitive fields (e.g. physics). When this issue was statistically controlled for, the data no longer demonstrated any gender differences (Albers, 2015; Volker & Steenbeek, 2015).

In sum, existing social barriers and challenges for women can implicitly hinder women from being equally successful in obtaining research grants, alluding to the fact that the grant funding peer review process is integrated within a bigger social and academic context. These issues imply that addressing extraneous variables beyond applicant gender in research that reflect these challenges is crucial to a better portrayal of the full picture of the situation. Nonetheless, research on grant funding peer review and decisions has thus far compared gender discrepancies simply at face value without reflecting much on these circumstances. Although some studies have suggested that the peer review process may not necessarily be skewed by reviewer biases during the evaluation process, they may not have sufficiently addressed gender disparities arising from the structural problems inherent in academia. To better understand how these problems work in the context of grant funding attainments, we need to acknowledge indices that reflect the societal challenges impacting final funding decisions and to identify the source of gender discrimination, be it at the level of the review process or on a larger structural level associated with academia (e.g. women to men ratio in senior positions in a specific institution, institutional family support). Stratifying datasets according to these critical variables such as application rate and applicants’ academic rank (Boyle et al., 2015; Holliday et al., 2014; Pohlhaus et al., 2011; Waisbren et al., 2008) as well as academic field, may thus prove to be insightful.

## Other potential sources and reasons for the mixed findings: methodological challenges in research

We have highlighted some potential sources leading to gender disparities in an effort to make sense of the mixed findings reported in past studies. However, we further argue that these mixed findings could also be attributed to the different methodological approaches observed across studies.

One critical issue that can result in causing such methodological discrepancies is that grant funding peer reviews do not necessarily have standardised criteria (Langfeldt, 2001). In other words, grant schemes and reviewers may have different benchmarks about what constitutes merit or being worthy of investment. In this manner, each peer review occurs somewhat on a case-by-case basis where each aspect or variable will exert varying degrees of influence within every review context. Therefore, the choices made to reflect the target characteristics of the grant scheme or the way the different variables will be operationalised in the reseach agenda depend heavily on the researchers’ decisions and the information made available to them. For instance, an applicant’s academic productivity, which reflects an applicant’s scholarly impact, has often been conceptualised through different bibliometric measures. Some studies have derived single normalised impact factors of publication counts, as Brouns (2000) did, whereas others have opted for measures that consist of indices. Tamblyn et al. (2018), for example, took bibliometric measures including the h-index and the sum of the impact factor of the applicant’s publications, but also the past funding experience, deriving a more comprehensive and composite index. Alternatively, some studies may not choose to consider productivity measures at all (e.g. Beck & Halloin, 2017; Burns et al., 2019), which could be due to a lack of access to such information, to a lack of perceived importance of such a factor, or even due to reasons relating to the feasibility of the statistical analyses. While failure to account for these co-occurring variables can lead to spurious results regarding the effect of applicant gender, it is simply not reasonable to account for all influencing factors in a tangible way. Indeed, in most studies, irrespective of their conclusions, efforts have been made to account for extraneous variables, although not exhaustively. The absence of standardisation in operationalising variables as well as the lack of uniformity in the inclusion criteria inevitably leads to variation across studies.

Relatedly, studies also differ in their study goals. Whereas some studies conduct simple examinations of gender disparities in funding *decisions* (e.g. Brouns, 2000; Titone et al., 2018; Zhou et al., 2018), others may examine the peer review *process* that leads to these decisions (e.g. Kaatz et al., 2016, 2015; van der Lee & Ellemers, 2015; Witteman et al., 2019). However, focusing on only one of these goals may occlude interactions between various variables contributing to the broader context, leading to the devaluation of women’s merit. Put differently, investigating only outcome inequities may illustrate some gender divide yet keep its sources unclear. Even when factors such as *performance* or *productivity* are accounted for, they may still not account for systemic biases, as discussed earlier.

To conclude, the variation observed across studies in the operationalisation of variables and research goals makes it difficult to consolidate our understanding on the issue of applicant gender and peer review, as each study can potentially be conceptualised as a singular or independent case (Guthrie et al., 2019; Ranga et al., 2012). The challenge is thus twofold. First, to better understand the multifactorial characterisation of grant funding peer review, research needs to address not only applicant gender, but all potentially affecting independent variables. Second, variation across studies is inevitable, as review contexts may differ across evaluations. This, however, may lead to studies operationalising specific concepts differently or setting distinct research goals, which may result in case-like-studies that are difficult to generalise.

## Discussion and conclusion

Throughout this paper, we have examined studies that investigated the potential impact of applicant gender on research grant peer review and funding decisions. On the one hand, a considerable amount of research demonstrates that gender discrimination in grant funding success is not empirically tenable. These studies have large sample sizes that afford reliability and typically include control variables. On the other hand, a host of researchers conducting grant specific, case-like studies have highlighted the impact of male-favouritism held by reviewers. Although these studies are less generalisable given their specificity, they nonetheless show that implicit gender biases have a sizeable impact on grant peer review and funding decisions within particular contexts (e.g. regarding specific disciplines or evaluation criteria). So, the question remains: are grant funding peer review and funding decisions biased against women?

Given the variability in the methodological approaches that researchers have employed to capture theses effects, the existing literature remains divided as to whether or not gender biases are integral in the grant funding peer review process and its decisions. The viewpoint we feature in this paper, however, is not intended to derive a definitive conclusion as to whether or not gender plays a role in impacting grant funding peer reviews and decisions per se. Instead, we argue that these two streams of findings are complementary and address the intricacy of the decision-making process, as well as the necessity of assessing women’s careers as a whole.

Whereas large-scale studies allude to a generally fair evaluation process, smaller-scale studies reveal that when certain contexts or assessment criteria are met, gender biases do in fact emerge. For instance, studies show that women are less favoured in cases where reviewers base their evaluations on the quality of the applicant as opposed to their scientific proposals (e.g. van der Lee & Ellemers, 2015; Witteman et al., 2019). These implicit gender biases that reviewers exercise may not necessarily skew the final funding decisions and thus, may remain undetected by studies even though prejudices may continue to have an indirect and persistent impact in the review process. Moreover, large-scale studies might rely more on data obtained by national funding institutions which might more or less have explicit gender policies in place. Therefore, the final funding decisions might not only be the result of the peer-review process (which seems more biased).

In this respect, future research should not simply pursue binary answers about the presence or absence of gender inequities but should aim to reveal the precise conditions in which potential biases arise. To pursue such goals, special attention should be given to clarifying the evaluation criteria and the order of their priority used to measure applicants’ performance, as well as to identify how each of these criteria may be prone to gender bias, at an individual, systemic, and/or societal level.

This is crucial, as we have argued that the impact of applicant gender cannot simply be evaluated by examining actual grant peer reviews and decisions alone. Essentially, the issue is fundamentally and deeply rooted in the academic culture and social structure. As opposed to men, simply being a woman in our androcentric culture brings about challenges that result in giving women fewer opportunities throughout their career and which have direct consequences for obtaining research grants (e.g. Pohlhaus et al., 2011; Waisbren et al., 2008). Future studies may need to shift their research focus to reflect on how gender is interwoven in academic settings and strive to better operationalise these implicit obstacles within their research agenda. In particular, elucidating how one’s life course and employment situation can indirectly impact academic performance will be essential, by addressing, for example, family situations, employment status (e.g. teaching load, precarity), and institutional support (e.g. research resources, strength of research promotion services). Additionally, while these issues go beyond the scope of this paper, it is important to note that beyond one’s gender, other potential diversity-related factors such as race, age, and religion may also bring about cumulative disadvantages. Better understanding the biases operating at different levels and recognising women’s career as an ensemble of variables is fundamental in achieving an amalgamated view of a complex phenomenon.

In essence, the potential challenges that female researchers face regarding grant funding are engrained at two distinct levels: biases that emerge during specific peer review contexts, and biases that can impact the researcher prior to the actual submission of the grant application. The issue, therefore, is complex and obtaining a more comprehensive idea of how these problems and variables interrelate and impact a woman’s career is not only fundamental in improving research on these issues, but also in adjusting the actual situation for female researchers in the future. Targeted actions are thus needed in practice within academia to resist the systematic biases that exist in the processes linked to research grant peer reviews. On an institutional level, strategies should be taken to remove structural and cultural obstacles that work against women. Steps could include measures that promote the retention of female researchers in academia, such as increasing the number of female faculty (i.e. appointing more women to higher positions within academia), relieving them of teaching and administrative responsibilities, or increasing permanent positions or funding schemes dedicated to female researchers. On an individual level, mentoring is shown to be effective in promoting both practical and emotional support in the academic context, leading participants to obtain greater research funding, higher publication rates, and better self-perceptions (Gardiner et al., 2007). Additionally, simply raising awareness and informing the younger generation of researchers about the issues is essential in changing attitudes that are deeply rooted. In terms of improving the system of grant peer review, training opportunities could be more actively introduced to refine the quality of reviewers’ assessments on inter-rater reliability (Sattler et al., 2015). Moreover, while difficulties may remain in anonymising applicants’ identities entirely, measures to implement a blinded review may result in providing more equal and reliable reviews (Bhattacharjee, 2012). Finally, political efforts also need to take shape to shift the status quo of institutional practices and trends.

To conclude, we have argued that multiple forms of gender inequalities persist and take form in society today which may indirectly impact critical academic indices. The factors underlying these differences may contribute to influencing the career trajectories of female researchers, and we encourage stakeholders to become well informed so as to take the necessary measures to improve common practices.

## Footnotes

1 Referring to Grade A staff (She Figures, European Commission, 2018).

2 In the current paper, the terms ‘grant peer review’ and ‘research grant funding decisions’ are used to refer to the processes related to the allocation of funding. Note that some of the issues may well be relevant within the more general notion of research funding or specific schemes such as fellowships.
